# When do the expectations of others matter? Experimental evidence on distributional justice and guilt aversion

**DOI:** 10.1007/s11238-020-09792-y

**Published:** 2020-12-22

**Authors:** Riccardo Ghidoni, Matteo Ploner

**Affiliations:** 1 Department of Economics, Management and Statistics, University of Milano-Bicocca, Milan, Italy; 2 Department of Economics, CentER, Tilburg University, Tilburg, The Netherlands; 3 Department of Economics and Management, University of Trento, Trento, Italy

**Keywords:** Justice, Guilt aversion, Entitlement rights, Beliefs, Experiment

## Abstract

Distributional justice—measured by the proportionality between effort exerted and rewards obtained—and guilt aversion—triggered by not fulfilling others’ expectations—are widely acknowledged fundamental sources of pro-social behavior. We design three experiments to study the relevance of these sources of behavior when considered in interaction. In particular, we investigate whether subjects fulfill others’ expectations also when this could produce inequitable allocations that conflict with distributional justice considerations. Our results confirm that both justice considerations and guilt aversion are important drivers of pro-social behavior, with the former having an overall stronger impact than the latter. Expectations of others are less relevant in environments more likely to nurture equitable outcomes.

## Introduction

A large body of literature has demonstrated that individuals are not only motivated by self-interest but also care about the consequences of their actions for others (e.g. Fehr and Schmidt 1999; Bolton and Ockenfels 2000; Charness and Rabin 2002). More recently, experiments have highlighted that also what others expect from us can influence the choices we make. Individuals tend to adjust their behavior not to let down others and avoid feeling guilty (see, among others, Baumeister et al. 1995; Charness and Dufwenberg 2006). However, according to social psychology literature, the emotion of guilt has a context-specific component, with some contexts being more conducive to guilt than others (Tangney 1992). Understanding under which circumstances the emotion of guilt plays an economically relevant role is an under-investigated issue.

Here, we conjecture that others’ expectations can be perceived as more or less *legitimate*, depending on the context faced by the decision-maker, and test whether decision-makers fulfill others’ expectations even when they clash with justice principles (see Bicchieri 2006, for a similar conjecture). We focus on a fundamental justice principle that motivates individuals to seek an equitable (proportional) allocation in terms of effort exerted to create an output and reward obtained for this effort (Konow 2000). This general distributional principle captures the essence of Locke’s *law of nature*, i.e. that property rights on goods originate in the effort exerted to generate them (Hoffman and Spitzer 1985). Our study investigates whether others’ expectations are more likely to be fulfilled when they are not in conflict with this acknowledged justice principle.

The interaction between guilt feelings and justice considerations might shape behavior in relevant economic interactions. Think, for example, of an employer who must choose between promoting an overconfident employee or an underconfident one. If the employees have similar performances, a guilt averse employer should give the promotion to the overconfident employee to minimize guilt for letting down one of the two employees. Similarly, if the best performing employee has (correctly) higher expectations of getting the promotion, this can further motivate a guilt averse employer to give the promotion to her. However, if the underconfident employee is the best performing one, the employer could give her the promotion, neglecting the employees’ expectations. Another example may come from charity giving. Think of donations to individuals who are facing the consequences of a natural disaster. Likely, a guilt averse individual will donate to meet the expectations of those in need. Yet, the emotional rush to give may be held back by considerations about potential corruption in the allocation process: if donations are likely to end in the wrong hands, even a guilt averse individual may refrain from giving.

We investigate the interplay between guilt and justice considerations in two distinct laboratory experiments. Study 1 builds on a modified dictator game where there is a probability with which a “lost wallet” is restored in the hands of the entitled owner, conditional upon the dictator choosing to return it. A returned wallet can also be misplaced by Nature to an unentitled recipient—who did not exert any effort to earn it—leading to an inequitable allocation. Only the dictator knows this specific, exogenous, restoring probability. Therefore, the entitled recipient cannot condition her expectation (and hence her disappointment for a missed return) upon the restoring probability. We communicate the entitled recipient’s expectation to the dictator to causally identify the effect of expectations. Moreover, we control for potential confounds linked to dictators’ self-serving biases by running a robustness check experiment that replicates the essential features of Study 1 but replaces the dictator with an external spectator with no material stake in the game (e.g., Almås et al. 2020). In Study 2, an external spectator must allocate a reward to one of two individuals that may differ in their expectations of being rewarded and in their desert, as captured by their relative productivity. Study 2 allows for a cleaner empirical identification than Study 1 and allows us to check the robustness of our conjecture across different setups.

In all our studies, simple guilt aversion predicts that decision-makers should try to fulfill expectations regardless of justice considerations (Battigalli and Dufwenberg 2007). According to our hypothesis, instead, they should be more likely to fulfill expectations when doing so also ensures a proportionality between effort exerted and rewards obtained. When fulfilling others’ expectations leads to a violation of justice principles, we expect optimistic expectations to become less relevant. In Study 1, returning the wallet to meet the optimistic expectations of the recipient may entail the risk of violating entitled ownership. In Study 2, meeting optimistic expectations may penalize the best performing worker. Thus, in both studies, expectations seem legitimate when they do not conflict with justice considerations based on effort-related entitlement.

While the literature on guilt aversion is rapidly growing, we are aware of only a few recent experiments that touch upon the issue of expectations’ legitimacy (Balafoutas and Fornwagner 2017; Pelligra et al. 2020). These studies focus on the nature of the requests made by recipients/trustees to dictators/trustors. When requests are too ambitious, they may not trigger guilt feelings because they are perceived as not legitimate. Another related study is the experiment by Danilov et al. (2018), who study the impact of descriptive norms and guilt feelings on giving in the dictator game. We share with these studies the attempt to refine the definition of guilt. However, our work differs from previous studies in the approach to expectations’ legitimacy. We adopt a widely acknowledged justice principle according to which outputs of the production should be allocated in proportion to individual inputs (Konow 2000), and define beliefs’ legitimacy in terms of accordance with this principle.

Our data show that both guilt aversion and justice considerations are key in driving allocation choices. Study 2 provides us with a direct assessment of the importance of the two sources and clearly shows that guilt is of secondary importance relative to justice. Furthermore, in contrast to our initial hypothesis, we do not identify any positive interaction between the two motivational sources. In fact, our studies show the opposite. Dictators in Study 1 and external spectators in Study 2 tend to neglect counterparts’ expectations when the distributional norm is clear, namely when the restoring probability is high in Study 1 and when a worker is better than the other. However, guilt aversion is still relevant in cases in which the distributional norm is less clear. These results are overall confirmed also by the robustness check of Study 1. In the concluding section, we discuss these findings and call for further research on the interaction between distributional norms and expectations.

The remainder of the paper is organized as follows. In Sect. 2, we position our contribution in both the literature on guilt aversion and on distributional justice. Sections 3 and 4 report design, hypotheses, and results for Study 1 and Study 2, respectively. General conclusions are discussed in Sect. 5.

## Literature review

Our paper contributes to the literature on the emotion of guilt in strategic interactions. Long-standing literature in social psychology has highlighted the role of guilt in shaping decision-making. Baumeister et al. (1994) stress how guilt can originate from actions causing harm to someone else. Individuals feeling guilty are more likely to engage in forms of pro-social behavior to compensate for the harmed party (Ketelaar et al. 2003; Nelissen et al. 2007). This literature has also documented that guilt can be experienced more in some contexts than in others (e.g. Tangney 1992). More recently, Charness and Dufwenberg (2006) and Battigalli and Dufwenberg (2007) developed a theory of guilt aversion—on which the present paper is focused—that models the decision-maker as averse to let down her counterpart. Specifically, a guilt averse decision-maker forms second-order beliefs on the first-order beliefs that the counterpart holds about the decision-maker’s behavior. Guilt is triggered by the counterpart′s disappointment, which is equal to the difference between the outcome she expected and the realized one. Several laboratory and field experiments provide support for this theory (e.g. Charness and Dufwenberg 2006; Bacharach et al. 2007; Dufwenberg et al. 2011; Bellemare et al. 2011; Babcock et al. 2015).

Some experiments cast doubts on the relevance of guilt aversion. Ellingsen et al. (2010) and Vanberg (2008) note that the positive correlation between the decision-maker’s choice and her second-order beliefs could be the result of a false consensus effect (Engelmann and Strobel 2000). To test for this hypothesis, Ellingsen et al. (2010) elicit the first-order beliefs of some subjects before the play and communicate them to the decision-makers. The authors do not detect any significant effect of more optimistic expectations on decision-makers’ choices in trust and dictator games. In a similar design, however, Reuben et al. (2009) find evidence of guilt aversion.

Theoretical models of guilt aversion à la Battigalli and Dufwenberg (2007) do not explicitly address the issue of beliefs’ legitimacy. One could conjecture that decision-makers only consider others’ expectations that they perceive as legitimate. Indirect support for this conjecture is given by Andreoni and Rao (2011). In their *Ask* treatment, a recipient can formulate a monetary request to the matched dictator. The authors report an interesting finding, labeled as “the paradox of obviousness”: when individuals ask for what is obvious, they obtain what they ask; when they ask for more than a fair share, they obtain nothing. More broadly, Bicchieri (2006) argues that individuals are more likely to follow a norm when others expect them to follow it, conditional upon others’ expectations being legitimate.

Our study also contributes to the literature on justice principles. In his extensive literature review, Konow (2003) highlights the importance of justice theories that relate fair allocations to individual actions. Equity theory (e.g., Adams 1963) provides clear guidance to assess the fairness of allocations in which a production stage is involved: an equitable allocation should preserve the proportionality of resources invested and rewards obtained across individuals. Thus, those investing more resources in the production of the output should obtain a larger share of it. Experiments in both social psychology and economics report empirical support for this justice principle (e.g., Leventhal and Michaels 1969; Mikula 1974; Konow 2000).

More recently, Cappelen et al. (2007) have identified three fairness ideals that dominate the debate about distributive justice: strict egalitarianism, libertarianism, and liberal egalitarianism. Strict egalitarianism defines justice in terms of equality of allocations, irrespective of the process leading to the production of wealth. Libertarianism and liberal egalitarianism define justice in terms of proportionality between inputs and outputs. The main difference is that libertarianism considers all inputs of the production process, while liberal egalitarianism only considers inputs that are under one’s own control (see also the accountability principle by Konow 1996). In our experiments, the libertarian and liberal egalitarian ideals overlap, as all production factors are controlled by the individual. Cappelen et al. (2007) classify most participants as either libertarian or liberal egalitarian, showing that the production phase has important implications for the allocation decision. The recent work by Almås et al. (2020) indicates that the large majority of participants in an allocation experiment take into account merit when choosing allocations and do not follow a pure egalitarian ideal.

## Study 1

### Experimental design

The modified dictator game: In a pre-stage game, two players work on a real-effort task to earn an endowment (wallet, henceforth). One of the two players is then selected at random to lose her wallet. The lost wallet is found by the other player. We refer to the player who lost her wallet as Entitled Recipient (*ER*) and to the one who found it as Dictator (*D*). The game also includes Nature and a third passive player who did not exert any effort and so has no initial endowment. We call the passive player Unentitled Recipient (*UR*).

**Fig. 1:**
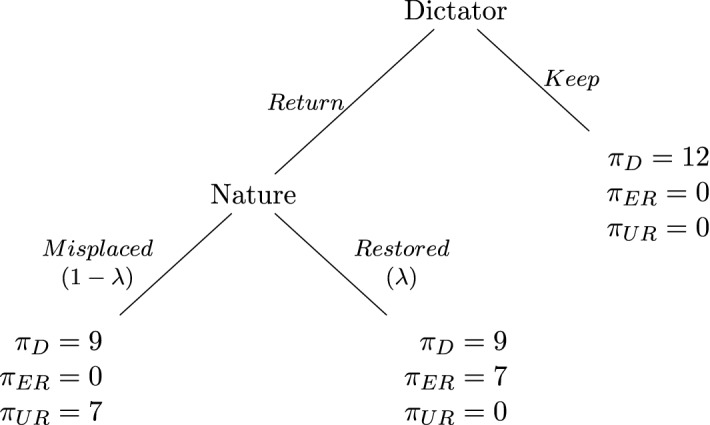
Modified dictator game

In our game (Fig. 1), *D* must choose between keeping *ER*’s wallet or returning it.^1^ If *D* keeps the wallet, the game ends with *D* owning her and *ER*’s wallets, while *ER* and *UR* get nothing. If *D* returns the wallet, the final outcome depends on Nature’s move: with probability $\lambda$, *ER* restores her wallet, and with probability $1-\lambda$, the wallet is misplaced to *UR*. More precisely, if *D* chooses Keep, she gets 12, and both *ER* and *UR* get 0. If *D* chooses Return, she gets 9, and either *ER* or *UR* gets 7, while the other gets 0, depending on Nature’s move. We sacrificed some realism in the payoff structure for two reasons.^2^ First, payoffs ensure a sizable disappointment for *ER* if she does not restore her wallet and had optimistic expectations about it. This way, the possible psychological cost of guilt for *D* is also sizable (see Sect. A in Appendix for details). Second, payoffs ensure that if *D* opts for Return, the level of restoring probability $\lambda$ neither affects the final efficiency nor inequality. Thus, if *D* is motivated by outcome-based social preferences, such as fairness (Fehr and Schmidt 1999; Bolton and Ockenfels 2000) or efficiency concerns (Charness and Rabin 2002), she should choose Return irrespective of the value of $\lambda$. The value of $\lambda$ is private information of *D*; *ER* and *UR* only know that $\lambda$ can take values 4/6, 5/6, or 6/6, with equal likelihood.^3^ As in Charness and Dufwenberg (2006), *ER* and *UR* do not observe *D*’s action. Thus, *ER* cannot infer whether *D* kept the wallet or Nature misplaced it.

**Fig. 2:**
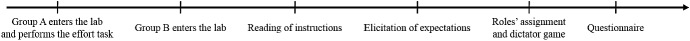
Timeline of the experimental session

The experimental session: Two groups of subjects participated in each session, group *A* and group *B*. Group *A* members actively participated in all stages of the experiment and played either in the role of dictators or entitled recipients (Fig. 2). Group *B* members, who acted as unentitled recipients, actively participated only in the beliefs’ elicitation stage and were free to surf the Internet during other stages.^4^

At the beginning of a session, group *A* members performed a real-effort task to earn their wallet. They had to count the number of zeros in seven $15\times 8$ tables containing 0 and 1 digits in random proportions, which sequentially appeared on their computer screens. For each table solved, they earned 1 token (1 token = €1). Subjects were not time-constrained and could make mistakes. At the end of the task, each group *A* member virtually owned a wallet of 7 tokens.^5^

After the task, group *B* also joined the session, and we read the instructions for the remaining stages of the experiment, i.e., the elicitation of expectations and the dictator game. To ensure a good understanding of the instructions (Bigoni and Dragone 2012), we complemented our instructions and software with illustrations, slides summarizing instructions, and control questions (see Sect. D in Appendix). The dictator game was repeated for three rounds, each time with a different $\lambda$ value (4/6, 5/6, or 6/6) in random order (randomized across sessions), unknown to *ER* and *UR*. Roles were fixed, and subjects were rematched after every round with a perfect strangers protocol. Only one of the three rounds was randomly drawn for the payment.

To rule out false consensus effects, we induced guilt feelings by providing *D* with *ER*’s first-order expectation (see Ellingsen et al. 2010, for a discussion). Before the game, we asked all members of groups *A* and *B* to state how many times out of the three rounds of the game they expected a generic *D* to return the wallet (Table 1). We rewarded subjects for the accuracy of their expectation through an incentive-compatible mechanism. At the end of the session, one choice of a dictator—different from the one used to pay the game—was randomly selected. If the selected choice was Return, the *more* optimistic, the stated expectation the *higher* the reward (computed via a quadratic scoring rule). Instead, if the choice was Keep, the *less* optimistic the expectation, the *higher* the reward. To avoid the omission of relevant information, before the elicitation, we informed group *A* members that their expectations could be disclosed to dictators during the game.^6^

**Table 1:** Elicitation of expectations

	Dictator will choose *Return*...			
	0 out of 3	1 out of 3	2 out of 3	3 out of 3
Your guess...	$\Box$	$\Box$	$\Box$	$\Box$
Your earnings if in the drawn choice...				
Dictator chose *Return*	€0	€2.80	€4.40	€5
Dictator chose *Keep*	€5	€4.40	€2.80	€0

Procedures: The experiment was programmed and conducted with z-Tree (Fischbacher 2007) at the Cognitive and Experimental Economics Laboratory (CEEL) of the University of Trento between April and September 2013. A total of 180 students took part in 12 sessions of 15 participants each (10 in group *A* and 5 in group *B*). Subjects were recruited via email using a dedicated software developed at CEEL.^7^ All subjects received a show-up fee of €3.

### Predictions and hypotheses

Standard theory predicts *D* to always choose Keep because $\pi _D(Keep) > \pi _D(Return)$. Outcome-based social preferences, like altruism (e.g. Cox et al. 2008), inequity aversion (e.g., Bolton and Ockenfels 2000; Fehr and Schmidt 1999), or efficiency concerns (e.g., Charness and Rabin 2002), can predict *D* to choose Return, but not to condition her choice upon the value of $\lambda$ or *ER*’s expectations.^8^ In contrast, the theories of equitable allocations (Konow 2000) and guilt aversion (Charness and Dufwenberg 2006) predict Return choices to depend upon the level of $\lambda$ and *ER*’s expectation, respectively.

When *D* finds *ER*’s lost wallet, an unfair and inequitable allocation is induced because *D* and *ER* have exerted the same effort, but *D* obtains (almost) all the surplus and *ER* obtains nothing. *D* can restore justice by returning the wallet to *ER*. Even though the final allocation in the case of a successful return does not equalize the *D* and *ER* payoffs, it reduces the striking disparity between inputs and outputs resulting from *D* keeping the wallet. Instead, if Nature misplaces the wallet to *UR*, an even less equitable allocation is in place: the wallet is given to someone who did not exert any effort to generate it. If the probability of misplacing the wallet is zero, a justice concerned *D* should return the wallet to prevent *ER* from ending up with no reward for her work. Instead, if the likelihood of misplacement is high, *D* will likely prefer to keep the wallet. This choice provides *D* with an extra reward relative to the return choice and avoids the double injustice of a misplacement: on the one hand, *ER* does not receive what deserved and, on the other hand, *UR* receives what is not deserved.^9^ So, for higher values of $\lambda$, *D* should be more likely to return the wallet, irrespective of *ER*’s expectation. This leads to our first testable hypothesis:

#### **Hypothesis 1**

(*Distributional justice*). For dictators aiming to preserve distributional justice, the likelihood of returning is increasing in the restoring probability ($\lambda$).

A guilt averse *D* experiences a psychological cost when letting *ER* down. The disappointment of *ER* is equal to the difference between what *ER* expected to obtain and her final payoff, i.e., zero in the case in which *D* keeps the wallet. *D* returns the wallet when the cost of guilt is large enough to overrule the material benefit of keeping it. In this respect, our belief elicitation presents a unique feature (see Table 1): *ER* cannot specify an expectation about *D*’s return decision for each value of $\lambda$. *ER* can only report the number of Return choices she expects from *D* over the three rounds of the game ($\beta \in \{0/3,1/3,2/3,3/3\}$). Hence, a higher $\beta$ should trigger the same degree of guilt in *D* irrespective of $\lambda$ (see Sect. A of the Appendix for a formal derivation). This leads to the following testable hypothesis:

#### **Hypothesis 2**

(*Guilt aversion*). For a guilt averse dictator, the likelihood of returning is increasing in the entitled recipient’s expectations of a return ($\beta$).

Our last hypothesis refers to the issue of beliefs’ legitimacy. Previous studies suggest that others’ expectations are effective in influencing behavior only when they are perceived as legitimate in the context faced by the decision-maker (Andreoni and Rao 2011; Bicchieri 2006; Balafoutas and Fornwagner 2017). We conjecture that a guilt averse decision-maker will perceive as illegitimate those expectations that would lead to taking an action that conflicts with justice considerations. More precisely, we test whether decision-makers are more likely to fulfill others’ expectations when doing so leads to an equitable distribution of the surplus. We expect a positive interaction between the restoring probability $\lambda$ and *ER*’s expectation $\beta$ on *D*’s decision to return the wallet: the positive impact of more optimistic expectations is strengthened by an institutional environment promoting equitable allocations; in contrast, when the institutional environment is weak, optimistic expectations are likely to be neglected. By the same token, if *ER* holds pessimistic expectations, *D* might not return even under a high $\lambda$ level because the cost of guilt is trivial. Thus, we formulate the following hypothesis:

#### **Hypothesis 3**

(*Guilt aversion with legitimate expectations*). For guilt averse dictators aiming to preserve distributional justice, the positive impact of an optimistic expectation ($\beta$) on the likelihood of returning is stronger when the restoring probability is higher ($\lambda$).

### Results

#### Expectations about dictator’s behavior

**Fig. 3:**
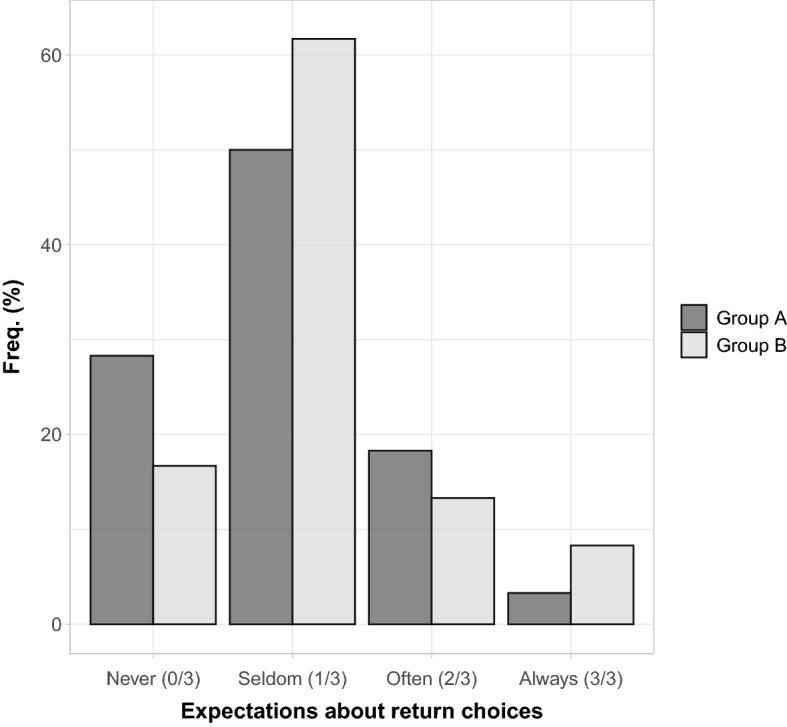
First-order expectations about dictator’s Return. The barplot shows the distribution of expectations over distinct return frequencies, from Never (0 returns in 3 rounds) to Always (3 returns in 3 rounds). As an example, the height of the bar in correspondence to Never captures the relative share of individuals believing that a generic dictator will never return the wallet. Different shades of gray identify the two groups in the experiment

Group *A* members knew that their expectations could be disclosed to dictators. Group *B* members, instead, knew that their expectations were not going to be disclosed to dictators. By comparing expectations of groups *A* and *B*, we can test for strategic manipulation of expectations by group *A* members. Figure 3 shows the distribution of the expectation about *D* return decisions for groups *A* and *B*. We adopt the labels “Never”, “Seldom”, “Often”, and “Always” to identify expectations that range from 0 returns out of 3 rounds to 3 returns out of 3 rounds.

Both in group *A* and group *B*, about 80% of the subjects expect *D* to choose to return in less than 2 out of 3 rounds, with the modal expectation corresponding to “Seldom” (1 out of 3). Although group *A* members are slightly more pessimistic than group *B* members, the two distributions are not statistically significantly different (Fisher’s exact test, $p=0.117$). We conclude that there is little evidence of expectations’ manipulation by group *A* members. Finally, we use earnings in the beliefs’ elicitation to measure beliefs’ accuracy (see Table 1). The median earnings in this task are equal to €4.40 for both *A* and *B* groups, just one step away from the maximum earnings of €5. Thus, beliefs are overall accurate in both groups.

#### Dictator’s decisions

Out of the 60 dictators, 28 (46.7%) never returned the wallet across all three rounds, and only 2 (3.3%) always returned it. Half of the subjects choose differently across rounds. This suggests that outcome-based social preferences are not a good fit to describe dictators’ behavior in our experiment. For a large share of dictators, both recipients’ expectations and restoring probabilities seem to affect return choices. When collapsing all levels of $\lambda$, the lowest percentage of returns (17.5%) is observed in correspondence of the most pessimistic expectation “Never”, in line with the guilt aversion prediction of Hypothesis 2. However, in contrast to Hypothesis 2, the highest percentage of returns is observed in correspondence to the intermediate expectation “Seldom” (29.6%), rather than to the more optimistic expectation “Often” (25.6%).^10^ When collapsing all expectations’ levels, consistent with Hypothesis 1 on justice considerations, the percentage of return choices is significantly higher for $\lambda =6/6$ (30.0%) than for lower levels of $\lambda$. Still, the percentage of returns for $\lambda =4/6$ and $\lambda =5/6$ is the same (21.7%).

**Fig. 4:**
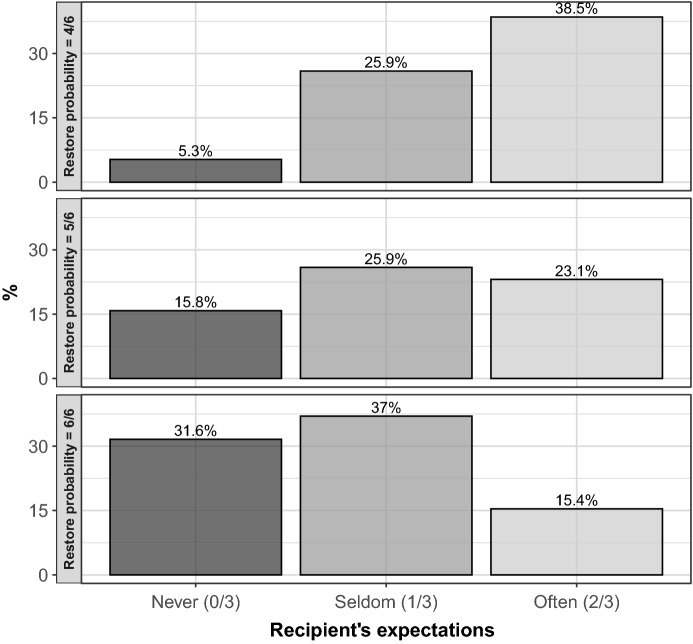
Return choices by recipient’s expectations and restoring probabilities. Each barplot provides a representation of the frequency of dictator’s return choices, in percentage terms, for different categories of recipient’s expectations ($\beta$), from Never to Often (Always is omitted because only one observation is available). As an example, the height of the bar in correspondence to Never captures the relative share of dictators returning the wallet when the recipient believes that a dictator will never return the wallet. Different panels capture different restoring probabilities ($\lambda$)

Figure 4 shows the joint effect of expectations and restoring probabilities, reporting the percentage of returns for alternative levels of $\lambda$ (rows) and *ER*’s expectation (columns). The percentage of returns monotonically increases in *ER*’s expectation only for $\lambda =4/6$ (upper panel), with a statistically significant increase in return choices between the expectation levels “Never” and “Often” (Fisher’s exact test, $p=0.029$). In contrast, for $\lambda =5/6$ and $\lambda =6/6$ the impact of expectations is non-monotonic, with no significant differences in return choices in correspondence to the expectation levels “Never” and “Often” ($p\ge 0.420$).^11^

### Regression analysis

The analysis reported above suggests that both restoring probabilities and entitled recipients’ expectations have a positive impact on return decisions, even though the effects are not fully in line with Hypotheses 1 and 2. Expectations and restoring probabilities seem to interact in shaping dictator choices. Here we present a regression analysis that casts light on the interaction between these behavioral drivers.

**Table 2:** Probit regressions on the determinants of Return choices

*Dep. var.:* Return (0, 1)	(1)		(2)		(3)	
Restoring probability ($\lambda$)	3.022** (1.165)		3.490** (1.218)		3.381** (1.272)	
Recipient’s expectation ($\beta$)	5.933* (2.459)		7.244** (2.553)		7.754** (2.820)	
Restoring probability $\times$ recipient’s expectation	− 6.712* (2.889)		− 8.162** (2.965)		− 7.622* (3.253)	
Dictator expectation					1.590** (0.586)	
Recipient’s expectation > Dictator expectation					− 0.886* (0.443)	
*Subject’s characteristics*						
Italian			− 0.559 (0.376)		− 0.506 (0.374)	
Male			− 0.270 (0.231)		− 0.288 (0.218)	
Experienced			0.184 (0.274)		− 0.184 (0.278)	
Working student			− 0.0325 (0.123)		− 0.0789 (0.107)	
_cons	− 3.345** (1.034)		− 3.266** (1.139)		− 3.577** (1.101)	
$N$	180		180		180	

Results from Probit regressions are reported. The unit of observation is a dictator’s choice. Individual-level clustered robust standard errors are in parentheses. $$^\circ p < 0.1$$, $$* p < 0.05$$, $$** p < 0.01$$, $$*** p < 0.001$$

Table 2 reports the outcomes of Probit regressions on the decision to return, controlling for repeated choices via clustered robust standard errors at the individual level.^12^ To test Hypothesis 3 of guilt aversion with legitimate expectations, we include as main covariates the restoring probability $\lambda$, the expectation of the entitled recipient $\beta$, and their interaction. In Model 2, we add a control for the subject’s characteristics collected at the end of the experiment. In Model 3, we investigate the impact of the dictator’s expectation and the relative standing of this expectation relative to that of the matched entitled recipient.^13^

All regressions show that higher restoring probabilities increase the likelihood of return, providing support to the relevance of justice considerations. At the same time, the positive and statistically significant coefficient of “Recipient’s expectation ($\beta$)” suggests that dictators facing more optimistic recipients are, on average, more likely to return. This provides support to guilt aversion.^14^ The coefficient of the interaction term is significant and negative. This runs against our Hypothesis 3, stating that the positive effect of the recipient’s expectation would be strengthened by a choice environment favoring an equitable outcome. The effect of optimistic expectations versus pessimistic expectations is thus weaker under higher restoring probabilities.

Finally, in Model 3, we find that the dictator’s expectation about the behavior of others in the same role is positively correlated to her decision to return (“Dictator expectation”). This pattern is compatible with false-consensus bias. Moreover, when the expectation of the entitled recipient is more optimistic than that of the dictator (“Recipient’s expectation > dictator’s expectation”), returns are less likely. A possible interpretation is that the counterpart’s expectations that are perceived as exorbitant relative to own expectations can discourage returns.

### Robustness check

When interpreting results from Study 1, we ascribe the drop in return rates for lower values of $\lambda$ to different justice assessments of final allocations by *D*. An alternative explanation could be the presence of a self-serving bias resulting from *D*’s exploitation of a “moral wiggle room” (e.g., Dana et al. 2007). This explanation might also rationalize the non-linear effect of $\lambda$, whereby the return rate drops substantially once uncertainty is introduced ($\lambda <1$), but not so when uncertainty increases further. We designed an additional experiment differing from Study 1 in a key aspect: return decisions are taken by an *external spectator* instead of *D*.^15^ Since the external spectator is paid a flat fee and has no monetary stake in the interaction, there is no scope for a self-serving bias to influence return decisions.

We recruited six participants to play in the roles of *D*, *ER*, and *UR* and 180 participants to play in the role of external spectators from the online platform Prolific.^16^ At the beginning of the experiment, we described to external spectators the setup of Study 1 up to the point where *ER* lost her wallet and told them that they were asked to take return decisions. We stressed that their decisions could be selected to actually pay participants playing the roles of *D*, *ER*, and *UR*. Each external spectator was randomly assigned to one of the three values of $\lambda$, leaving us with 53 spectators facing $\lambda =4/6$, 62 facing $\lambda =5/6$, and 65 facing $\lambda =6/6$. Thus, $\lambda$ was experimentally manipulated in a between-subject fashion, differently than in Study 1. Moreover, external spectators were asked to take four return decisions, one for each possible first-order belief level of *ER* about the decision of a generic external spectator to choose to return (*strategy method*; see Bellemare et al. 2018, for a discussion in the context of guilt aversion).^17^ This element of the design differs from Study 1, where *D* participants experienced different combinations of $\lambda$ and expectations at random, but allowed us to collect a more balanced number of return choices for each combination of *ER*’s expectation and $\lambda$ values. Once the data collection was complete, we randomly selected two external spectators and matched each of them with a triplet, including *D*, *ER*, and *UR* participants. We paid these participants according to the spectator’s return decision, corresponding to the actual expectation level stated by *ER*. More details on this experiment are in Sect. B of the Appendix.

**Fig. 5:**
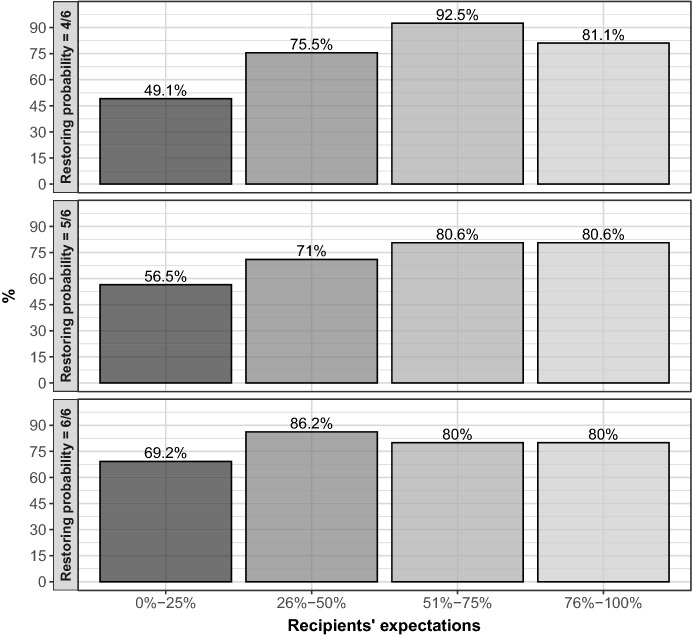
Return choices by recipients’ expectations and restoring probabilities—Robustness check. Each barplot provides a representation of the frequency of return choices, in percentage terms, for different categories of first-order expectations of recipients ($\beta$), from 0–25% to 76–100%. As an example, the height of the bar in correspondence to 0–25% captures the relative share of external spectators returning the wallet when the recipient believes that a generic eternal spectator is very unlikely to return the wallet. Different panels capture different restoring probabilities ($\lambda$)

Figure 5 shows the joint effect of *ER*’s expectations and restoring probabilities on return choices. The percentage of returns is plotted by expectation categories. Each panel refers to a different restoring probability. The percentages of returns are generally higher than those observed in Study 1 (see Fig. 4). However, the overall impact of $\beta$ and $\lambda$ are comparable across the two studies. Expectations have a positive impact on return choices, but their effect is not strictly monotone. As in Study 1, expectations appear to play a more important role under $\lambda =4/6$. Restoring probabilities also affect return choices positively, especially under more pessimistic expectations. Regression results are in line with those of Study 1 (see Table 2), confirming the positive and statistically significant effects of $\beta$ and $\lambda$ ($p<0.05$, see Table 4 in Appendix), as well as the negative interaction between the two dimensions ($p<0.05$).

To improve our understanding of the determinants of return choices, we also asked external spectators about the social appropriateness of choosing to return on a four-point Likert scale, given each *ER* expectation level and the specific restoring probability they had faced. For each spectator, we then selected at random one of the four submitted evaluations. Participants knew that if the selected evaluation corresponded to the modal evaluation in the experiment (given the same $\lambda$ level), they would earn an additional bonus of 0.50 GBP. This incentive scheme, based on a coordination game, has been introduced by Krupka and Weber (2013) to identify social norms in experimental games. We find that both expectations and restoring probabilities affect the perceived social norm in the same direction of choice data: lower levels of the two factors are associated with lower levels of perceived social appropriateness (a detailed analysis is available in Sect. B.2 of the Appendix). This suggests that the two justice factors not only impact on allocation choices but also on the perception of social norms.

## Study 2

The robustness check experiment mitigates the concern of a self-serving bias driving the results of Study 1. Yet, Study 1 and its robustness check are not immune to some other caveats that could hamper a clean identification of justice concerns. The payoffs’ structure could be somewhat confusing to participants (see footnote 2 for a discussion). Moreover, it remains questionable that the least preferred outcome for a *D* motivated by restoring justice is truly the one where the *UR* gets the wallet. We hence implemented a second experiment where these caveats are absent. Furthermore, Study 2 helps to corroborate the evidence from Study 1 by testing Hypotheses 1–3 in a different setup.

### Experimental design

The experiment inspired by Almås et al. (2020) includes two types of sessions: the Worker sessions and the Spectator sessions. Below, we describe them in detail.

Worker session: Participants in the Worker session had 5 min to work on a task, which consisted of solving as many sums as they could. They had to add up five numbers of two digits each; all digits were randomly generated at the individual level. A worker only moved to the next problem once she entered the correct solution to the current problem. Following Almås et al. (2020), participants were not informed before the task of the precise payment scheme. They only knew that they would receive €3 and that they could earn additional money through their actions and the actions of other participants. Moreover, we made clear that we would record their effort and that they received one point for every correct answer.

After the task, we informed participants that we would form pairs of workers and that an external third party (i.e., the spectator) would have to assign additional €6 to one of the two workers. They were also informed that the spectator would know who in the pair was the most productive worker and that their identity would remain anonymous. Finally, we asked workers to state their expectations. We asked first with which probability they expected to be the worker in the pair with the highest number of correctly solved problems. We then asked them to state their expectation of being selected by the spectator to receive the additional €6. This latter expectation is our proxy of the worker’s first-order beliefs about the spectator’s behavior.

Since first-order beliefs are key for the identification of guilt aversion, we gave workers an incentive to truthfully report them via a quadratic scoring rule.^18^ Workers were informed that their expectations could be disclosed to the spectator. We did so to avoid the omission of relevant information.

Spectator session: Spectators were paid a flat fee of €7 and were provided with a brief description of the Worker session. Next, they were individually presented with 20 pairs of workers. For each pair, every spectator had to choose to which of the two workers to assign €6.^19^ Alternatively, spectators could choose to assign the money to one of the two workers selected at random via a virtual coin flip.^20^ When making her decision, the spectator only knew who was the most productive worker in the pair and who between the two workers had the highest expectation of obtaining the reward. We opted for this binary information to simplify our analysis, as it is robust to outliers and does not require to control for the distance between workers’ productivity and expectations. Given this choice constraint, spectators could not reward workers in exact proportion to their input, but could still decide to minimize the distance between inputs and outputs by rewarding the most productive worker. Taking into account that two workers may have the same expectation and the same productivity, we face nine possible combinations of relative performance and relative expectation.^21^

At the end of the session, we randomly selected one spectator, and all 20 pairs of workers were paid according to the allocation decisions of the selected spectator. Before the payment, we asked all spectators to fill the Test of Self-Conscious Affect (henceforth TOSCA-3, Tangney et al. 2000) to gather a more direct measure of their guilt sensitivity (Bellemare et al. 2019).^22^

Procedures: We first conducted two Worker sessions of 20 subjects each, and then three Spectator sessions of 20 subjects each. Participants were ex-ante unaware of whether they signed up for workers’ or spectators’ sessions. All sessions were conducted at CEEL using z-Tree (Fischbacher 2007). Instructions are in Sect. C.1 of the Appendix.

### Predictions and hypotheses

The third party is an external spectator who has no material stake in the reward assignment but can still suffer psychological costs when disappointing the workers’ expectations and/or violating distributional justice principles. Therefore, while the experimental setup is different form Study 1, Hypotheses 1–3 can be reiterated in Study 2 as well. An important difference between the two studies lies in the definition of the entitlement rights to the reward. In Study 1, all participants knew who the entitled recipient was, while in Study 2, workers could only conjecture about their merit to be rewarded when stating their expectations.

Since spectators in Study 2 know the relative performance of the two workers, we can reformulate Hypothesis 1 of Study 1: spectators aiming at preserving distributional justice should assign the reward to the worker who showed to be more productive. Higher productivity is taken here as a proxy of higher investment (input) in the production phase that, according to our general justice principle, calls for higher rewards (output). Support to this conjecture also comes from Almås et al. (2020), who show that the majority of participants in their experiment rewarded the more productive workers.

Differently than in Almås et al. (2020), spectators were also informed about the workers’ relative expectations of receiving the reward from the spectator. These expectations are our proxy for the worker’s first-order beliefs about the behavior of the spectator. As in Study 1, we assume that by communicating the workers’ relative first-order beliefs to the spectator we can exogenously manipulate the spectator’s second-order beliefs, which represent the source of guilt feelings. Hence, we can test the relevance of expectations in the same vein of Hypothesis 2 in Study 1: if the spectator is guilt averse, the worker with higher expectations to obtain the reward should be the one to obtain it.

We are particularly interested in conditions that create tension between productivity and expectations. Situations in which a worker in the pair has higher (lower) expectations and the other worker performed worse (better) are key to test our hypotheses on beliefs’ legitimacy. Following the line of reasoning of Hypothesis 3 in Study 1, we should observe a positive interaction effect on the probability of receiving the reward when a worker has both the higher productivity and the higher expectation. Indeed, optimistic expectations of obtaining the reward are legitimate in this setting only when they are matched by higher productivity, given that beliefs originate in the subjective expectation of being the most productive worker in the pair.

### Results

#### Workers’ expectations

Our data reveal that workers are generally well-calibrated in their expectations, with a median reported expectation of 50% for both the probability of being the most productive in the pair and for the probability of being rewarded by the spectator. The overall soundness of expectations is also confirmed by the strong correlation between the beliefs about one’s own relative productivity and actual productivity (Spearman’s rank correlation $\rho = 0.764$, $p<0.001$).

The comparison of beliefs about being rewarded and being the most productive in the pair allows us to gain an insight into workers’ perception of the criterion adopted by the spectator to assign the reward. In line with our design assumptions, the two sets of beliefs are positively correlated (Spearman’s rank correlation $\rho = 0.670$, $p<0.001$). Thus, the expectation of being rewarded seems mainly driven by the belief about the relative productivity in the pair, with those who believe to be more productive entertaining higher expectations of being rewarded.

#### Spectators’ choices

Out of the 1200 spectators’ choices we collected, only 9.8% are associated with the use of the random device to allocate the reward. The use of the random device is mainly associated with choices in which the two workers are not distinguishable in terms of relative performance and/or expectations (80.2%). The following analysis focuses only on actively expressed choices, for random choices carry no informative content.

**Fig. 6:**
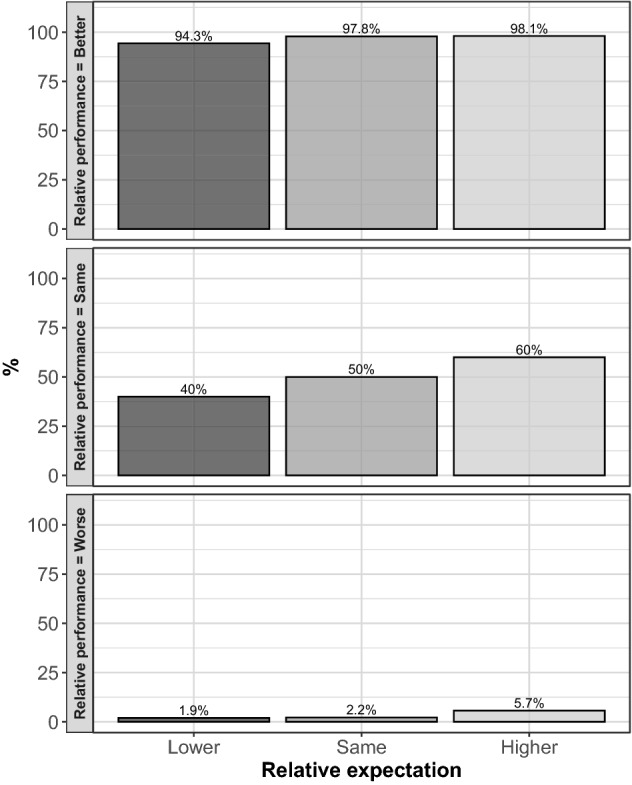
Reward choices by relative efforts and expectations. Each barplot provides a representation of the relative frequency, in percentage terms, of reward choices for different relative expectations of recipients, from lower to higher. As an example, the height of the bar in correspondence to lower captures the share of workers being rewarded when they have lower expectations than those of the other worker they are matched with. Different panels capture different levels of relative performances

Figure 6 shows the frequency of reward allocation for different combinations of relative performance and relative expectation. When a worker solved fewer (more) problems than the matched subject, her relative performance is equal to *Worse (Better)*. When the two workers solved the same number of problems, the relative performance is equal to *Same*. Similarly, when a worker has higher expectations about receiving the reward, her relative expectation is equal to *Higher (Lower)*. When the two workers have the same expectation, the relative expectation is equal to *Same*.

The overall probability that a worker with a better relative performance obtains the reward is equal to 97.2%, while the overall probability for a worker with higher relative beliefs is equal to 72.5%. Figure 6 shows that, when combining the two dimensions, the highest likelihood of obtaining the reward is observed when a worker has both higher expectations and better performance (98.1%). A better relative performance strongly increases the likelihood of receiving the reward relative to both the same and worse levels for all relative expectation levels. This finding provides support to the distributional justice hypothesis (Hypothesis 1). A similar pattern is qualitatively observed also for higher expectations, though the effect is much more moderate than for relative performance. The marginal impact of different relative expectation levels is very small with reference to workers who display better performance.^23^ Thus, the guilt aversion hypothesis (Hypothesis 2) is only moderately supported by our data.

Non-parametric tests show that there is little difference in the likelihood of being rewarded for different levels of relative expectations, given the level of relative performance (Wilcoxon signed rank test, $p \ge 0.083$).^24^ In contrast, when keeping fixed the level of relative expectations, higher levels of relative performance significantly increase the likelihood of being rewarded (Wilcoxon signed rank test, $p \le 0.005$). A full assessment of the main effects of our treatment variables and thier interaction is provided in the regression analysis below.

#### Regression analysis

Table 3 reports the outcome of Probit regressions, controlling for repeated choices via individual-level clustered robust standard error. The dependent variable *Rewarded* is equal to 1 for the worker rewarded by the spectator and equal to 0 for the unrewarded worker. As explanatory variables, we consider the relative performance and the relative expectation of the worker to whom the choice of the spectator refers. Specifically, *PerfBetter* is equal to 1 when the worker performed better than the matched worker, and equal to 0 otherwise. *ExpHigher* is equal to 1 when the worker has a higher expectation of receiving the reward than the matched worker, and equal to 0 otherwise.^25^ Finally, *GuiltAverse* is a measure of guilt aversion obtained from the TOSCA-3 questionnaire. Specifically, if a subject obtains a score in the questionnaire equal or greater than the median score, the variable has value 1, otherwise it is equal to 0.^26^ In Model 1, we consider only the main effects of the performance and expectation variables. In Model 2, the interaction between the two variables is also considered. Finally, in Model 3, we control for guilt sensitivity and for its interaction with the beliefs of the counterpart.

**Table 3:** Probit regressions on the determinants of reward assignment

*Dep. var.:* Rewarded (0, 1)	(1)		(2)		(3)	
PerfBetter	3.391*** (0.250)		3.605*** (0.255)		3.612*** (0.254)	
ExpHigher	0.387$^\circ$ (0.200)		0.567** (0.216)		0.435 (0.275)	
PerfBetter $\times$ ExpHigher			− 0.462* (0.218)		− 0.469* (0.221)	
GuiltAverse					− 0.158 (0.183)	
ExpHigher $\times$ GuiltAverse					0.262 (0.362)	
_cons	− 1.681*** (0.126)		− 1.752*** (0.134)		− 1.672*** (0.176)	
Observations	1082		1082		1082	

Results from Probit regressions are reported. Individual-level clustered robust standard errors are in parentheses. $$^\circ p < 0.1$$, $$* p < 0.05$$, $$** p < 0.01$$, $$*** p < 0.001$$

The regression outcomes of Table 3 confirm the strong impact of a better performance in increasing the likelihood of receiving the reward. A positive and significant impact is also observed for higher expectations, even though the effect is only marginally significant and much smaller than that estimated for the measure of relative performance.^27^ Model 2 shows that the two measures taken into account register a significant negative interaction: the effect of optimistic expectations is weaker when merit is salient.^28^ Thus, the evidence runs against our Hypothesis 3, similar to Study 1. The effect is also observed when controlling for guilt aversion of the decision-maker, but the impact of expectations becomes statistically not significant (Model 3).

## Conclusions

Several experiments have shown that decision-makers tend to be averse to let others down to avoid guilt (Charness and Dufwenberg 2006). We contribute to the literature by studying how decision-makers react to expectations that they may or may not perceive as legitimate, given the choice environment they face. Relying on previous evidence in the literature, we conjecture that decision-makers are more likely to fulfill their counterpart’s expectation when this is perceived as legitimate, i.e. when it is in line with the decision-maker’s justice considerations (Konow 2000).

In Study 1, we argue that a dictator could perceive as legitimate an optimistic expectation when such expectation is not at odds with justice principles based on the proportionality between effort exerted and rewards obtained. Similarly, in Study 2, the legitimacy of expectations is assessed against the relative performance of two workers in a simple task. An external spectator could perceive as legitimate a worker’s expectation to be rewarded only when such a worker performed better than a competing worker.

Results from our studies generally support the hypotheses that others’ expectations and justice considerations are important drivers of decision-making. However, in Study 2, the impact of expectations seems weaker than in Study 1. This may be explained by the different institutional settings that nurture the beliefs of the interacting parties. In Study 2, they are merely based on the subjective expectation of being the most productive worker in the pair. Thus, the external spectator may have primarily focused on the objective measure of productivity and only secondarily on others’ expectations. Differently, in Study 1, the expectations of an individual who lost her wallet are grounded in an objective measure of entitlement (having worked to earn the wallet). Thus, they may be more relevant for decision-makers, especially when justice consequences are more ambiguous.

Our results unanimously suggest that others’ expectations are more salient in some contexts than others (Tangney 1992). However, contrary to our initial conjecture, guilt aversion and justice considerations do not reinforce each other. Decision-makers tend to give less weight to their counterparts’ expectations when it is clear how to enforce distributional justice. In Study 1, when the restoring probability is high, dictators return the wallet to the entitled owner even if she holds pessimistic expectations. A similar result is obtained in a robustness check where the decision-maker had no stake in the interaction, thus ruling out the confound of a self-serving bias triggered by the exploitation of moral wiggle rooms (Dana et al. 2007). In Study 2, external spectators tend to reward the best worker even if she is relatively more pessimistic than the other one. Decision-makers rely more on others’ expectations when the risk that distributional justice will be (exogenously) violated is high or when merit is unclear.

We believe that this result may deserve further attention by future research as it provides stimulating insights into the working of distributional norms. Data collected suggest that when the distributional norm is clear, descriptive expectations of others become almost irrelevant for the decision-maker, which will likely follow the norm in any case. Instead, when the distributional norm is less clear, individuals may rely more on a subjective measure of justice, as captured by the counterpart’s expectation, which triggers a sense of guilt when disappointed.

## Appendix

### A Derivation of Hypothesis 2 for Study 1

A guilt averse *D* who chooses to keep the wallet obtains a material payoff of 12 but suffers a cost of guilt increasing in *ER*’s disappointment. The disappointment of *ER* is measured by the difference between what *ER* expected to obtain and her final payoff, which is 0 in the case in which *D* keeps the wallet. Instead, a guilt averse *D* who returns the wallet will obtain a lower material payoff of 9, but his sense of guilt will be null because he did his best to fulfill *ER*’s expectation. So, *D* returns the wallet only if the cost of guilt is large enough to overrule the material benefit of keeping *ER*’s wallet, i.e. when $12-\theta \bigl (E_{ER}(\pi _{ER})-0\bigr ) > 9$, where $\theta >0$ is *d*’s guilt sensitivity and $E_{ER}[\pi _{ER}]$ is the payoff *er* expected, which is equal to

1 $$\begin{aligned} E_{ER}[\pi _{ER}]&=\frac{1}{3}\beta _{\lambda =4/6}\left( \frac{4}{6}7+\frac{2}{6}0\right) +\frac{1}{3}\beta _{\lambda =5/6}\left( \frac{5}{6}7+\frac{1}{6}0\right) +\frac{1}{3}\beta _{\lambda =6/6}\left( \frac{6}{6}7+\frac{0}{6}0\right) = \nonumber \\&= \frac{7}{18}\left( \beta _{\lambda =4/6}{4}+\beta _{\lambda =5/6}{5}+\beta _{\lambda =6/6}{6}\right) \ , \end{aligned}$$

where $\beta _{\lambda }\in \{0,1\}$ is the probability with which *ER* expects *D* to return given a certain value of $\lambda$. Two important features of the belief elicitation must be taken into account. First, we restrict the belief space: subjects can only state how many times out of three interactions they expect a generic *D* to return the wallet (Table 1). So, $\beta _{\lambda }$ can only take value 0 (when *D* is expected to keep) or 1 (when *D* is expected to return). Second, subjects cannot specify an expectation $\beta _\lambda$ for each value of $\lambda$. Instead, they can only indicate the generic number of returns they expect from *d* over the three rounds, i.e. $\beta =\beta _{\lambda =4/6}+\beta _{\lambda =5/6}+\beta _{\lambda =6/6}$ (with a slight abuse of notation). These two features of the belief elicitation are key to keep distinct guilt aversion and justice considerations. In particular, for higher $\beta$, a guilt averse *D* is always more likely to choose to return irrespective of the faced $\lambda$.

To derive the minimum guilt sensitivity threshold ($\theta ^*$) necessary to induce *D* to return the wallet, we would need to assume that *ER* deems more likely that *D* returns the wallet when $\lambda$ is higher. As an example, when $\beta =1/3$ *ER* expects *D* to return only when $\lambda =6/6$, when $\beta =2/3$ *ER* expects a return for $\lambda =5/6$ and 6/6, and so on. However, it is important to remark that Hypothesis 2 still holds also under the more general assumption that $dU(Keep)/d\beta <0$.

### B Additional details on the robustness check of Study 1

#### B.1 Experimental design and procedures

The experiment consisted of two separate sessions: the worker session and the external spectator session. The worker session closely followed the procedures of Study 1, with only a couple of noteworthy differences. First, *D* was no longer asked to take the return decisions; this task was instead assigned to external spectators. Second, *ER* was no longer asked to estimate the number of return decisions by *D*, but the likelihood with which a generic external spectator would choose to return. We incentivized the elicitation of *ER* beliefs with a quadratic scoring rule very similar to the one used in Study 1. *ER* could choose among four probability intervals: 0–25%, 26–50%, 51–75%, or 76–100%. We constrained beliefs into four categories for comparability reasons with Study 1. Also, for comparability reasons, we chose to keep the same payoff structure of Study 1.

The spectator session was divided into two separate parts. Participants did not know the content of each part in advance. In part 1, we briefly described the worker session to external spectators and told them that they had to choose whether or not to return the lost reward to *ER*. For this task, they received a flat fee of 1.06 GBP. External spectators were randomly assigned to one value of the restoring probability $\lambda$ equal to 4/6, 5/6, or 6/6. To avoid creating multiple sessions for the same experiment on Prolific, which could lead to the risk of sorting, the software implemented a true randomization of $\lambda$ at the individual level. As a consequence, we collected slightly fewer observations for $\lambda =4/6$ (*N* = 53) than for $\lambda =5/6$ (*N* = 62) and $\lambda =6/6$ (*N* = 65). We asked the external spectators to make four return choices, one for each level of the first-order beliefs of *ER* (strategy method).

In part 2, participants were asked to evaluate the social appropriateness of choosing to return, given each level of *ER* belief and the value of $\lambda$ faced in part 1. Participants could obtain a bonus payment of 0.50 GBP for part 2 if one of their four evaluations selected at random matched the modal evaluation across all participants facing the same $\lambda$ and belief level. This incentive scheme generates a coordination game, and it has been proposed by Krupka and Weber (2013) to identify social norms in experimental games.

Worker and external spectator sessions took place between June 12 and 13, 2020, on the online platform Prolific (https://www.prolific.co/). We restricted participation to students between 18 and 30 years old and who accessed the experiment using either a laptop or a computer (smartphones were not allowed). Each subject could only participate once in the experiment, either in the worker session or in the external spectator session. The experiment was programmed using oTree (Chen et al. 2016). To adapt the experiment to an online setup, we streamlined instructions compared to Study 1, and we omitted the quiz on instructions. Furthermore, thanks to the strategy method used for beliefs in the external spectator session, we were able to circumvent a real interaction between workers and external spectators. For simplicity, we first implement the worker session and then the worker session. Six subjects were recruited for the worker session, and 180 subjects were recruited for the spectator session. Both sessions lasted roughly 10 min on average. Each token earned in the worker session was converted to 1 GBP (the currency used on Prolific).

### B.2 Empirical analysis

Table 4 reports the results of a regression analysis about the decision to return, controlling for repeated choices via clustered robust standard errors at the individual level. The dependent variable is the decision of an external spectator to return the wallet ($1=$ Return, $0=$ No return). The main explanatory variables are the restoring probability ($\lambda$), *ER*’s expectation ($\beta$), and their interaction. Furthermore, we control for gender and age of the external spectator. Both restoring probability and beliefs have a statistically significant, positive impact on the decision to return. Instead, the interaction term has a negative and significant sign. These regression outcomes corroborate the results reported above for Study 1 (see Table 2, model 2).

**Table 4:** Probit regressions on the determinants of Return choices

*Dep. var.:* Return (0, 1)	
Restoring probability ($\lambda$)	2.232 (0.981)*
Recipient’s expectation ($\beta$)	0.900 (0.321)**
Restoring probability $\times$ recipient’s expectation	− 0.800 (0.377)*
*Subject’s characteristics*	
Age	0.025 (0.020)
Male	0.108 (0.125)
_cons	− 2.224 (0.971)*
$N$	720

Results from Probit regressions are reported. The unit of observation is the choice of an external spectator. Individual-level clustered robust standard errors are in parentheses. $$^\circ p < 0.1$$, $$* p < 0.05$$, $$** p < 0.01$$, $$*** p < 0.001$$

Table 5 summarizes the outcomes of the social norms elicitation à la Krupka and Weber. For each restoring probability and *ER*’s expectation, we report the frequency of external spectators who deemed the decision to return in that condition “Very inappropriate” (– –), “Somewhat inappropriate” (−), “Somewhat appropriate” ($+$), and “Very appropriate” ($++$). The modal value is identified in italics. Following Krupka and Weber (2013), we also report a *score* of social appropriateness computed by assigning value − 1 to – –, − 1/3 to −, 1/3 to $+$, and 1 to $++$ and computing the average. The lowest score of social appropriateness is detected for the lowest restoring probability level ($\lambda =4$) and the lowest expectation level ($\beta$ = 0–25%), with 38% of the participants deeming return as “Very inappropriate”. At the other end of the spectrum, we find the score of social appropriateness in correspondence to the highest restoring probability ($\lambda =6$) and the highest expectation level ($\beta$= 76–100%), with 72% of the participants deeming return as “Very appropriate”. Thus, both beliefs and restoring probabilities seem to affect the perceived social norm, in the same direction identified by choice data. At a more detailed level, social appropriateness scores increase in expectation levels, but for 76–100% for $\lambda =4/6$. Social appropriateness also generally increases in the restoring probability but for the intermediate expectation level 51–75%.

**Table 5:** Relative frequency of social appropriateness evaluations

Restoring probability ($\lambda$)	B’s beliefs ($\beta$)	– –	–	$+$	$++$	Score
4/6	0–25%	*38*	23	26	13	− 0.233
5/6	0–25%	*27*	*27*	*27*	18	− 0.097
6/6	0–25%	23	*31*	25	22	− 0.036
4/6	26–50%	6	28	*47*	19	0.195
5/6	26–50%	3	37	*44*	16	0.151
6/6	26–50%	6	28	*48*	18	0.190
4/6	51–75%	4	9	42	*45*	0.522
5/6	51–75%	3	18	*45*	34	0.398
6/6	51–75%	3	18	*45*	34	0.395
4/6	76–100%	19	9	19	*53*	0.371
5/6	76–100%	15	10	10	*66*	0.516
6/6	76–100%	14	5	9	*72*	0.600

To test for differences in social appropriateness across alternative restoring probabilities, we compute the average score of appropriateness at the individual level for different expectation levels and a given $\lambda$. A series of Wilcoxon rank-sum tests comparing averages across $\lambda$ levels shows that there are no significant differences in scores ($p>0.27$). To test the impact of expectations, we pairwise compare scores for different belief levels across different $\lambda$ levels. A series of Wilcoxon signed-rank tests shows that all differences between belief levels are statistically significant ($p<0.044$). These results are also confirmed by an ordinal logistic regression with random effects at the individual level (available upon request), showing that the explanatory variable capturing belief levels significantly and positively affects social appropriateness assessment while restoring probability has no statistically significant impact. However, when assessing these results, it must be taken into account the different nature of the elicitation of $\lambda$ and $\beta$. The latter is obtained in a within-subject setting that might have prompted an “ordering” of evaluations by the same individual, while the former is obtained from distinct individuals.

### C Instructions

#### C.1 Study 1

*The first 10 participants enter the lab.*

Welcome!

For showing up you earn €3. During the session you can earn more money. The total amount will be paid to you in private at the end of this session. Please, follow the instructions carefully and do not speak to the other participants. If you have questions, raise your hand and one of the experimenters will answer to you in private.

The session consists of four stages. You are identified as the *Green Group* and you are going to complete the first stage. After having completed the first stage, another group of participants will enter this room, and will be identified as the *Red Group*.

Stage 1—Work. In this stage you must count the number of zeros in seven tables that will consecutively appear on your screen. For each table you will earn 1 token, once you input the correct number of zeros.

In a following stage, something unforeseen could occur which implies the loss of the tokens you have earned. At the end of the session, each token you own will be converted into €1. *The experimenter stops reading instructions, and the green group preform the task.*

The Red Group can now enter the lab.

*The red group enter the lab, and the experimenter restarts reading the instructions.*

Welcome!

For showing up you earn €3. During the session you can earn more money. Please, follow the instructions carefully and do not speak to the other participants.

The 10 participants who were already in this room are identified as the *Green Group*. Instead, the five participants that have just entered are identified as the *Red Group*.

The members of the Green Group have already been here for about 15 min, and have just completed the Stage 1 of this session, which consisted of counting the number of zeros in several tables. For this task, every Green Group member has earned 7 tokens. At the end of the session, each token you own will be exchanged with €1.

The Red Group members will only participate in the next Stage of the session, and then they will be allowed to surf the Web. Instead, the Green Group members will have to actively participate throughout the session. Before being allowed to leave, they will have to wait until all the Red Group members are paid.

In order to understand the Stage 2 (*estimation*) you need to learn about Stage 3 (*unforeseen event*) and Stage 4 (*decision*). Before proceeding to Stage 2, we anticipate you the instructions for Stages 3 and 4. *Everyone,* please follow carefully these instructions.

Stage 3—Unexpected. In this stage, participants do not take any decisions. The computer will implement all the procedures automatically.

If you are a Green Group member, you could suffer an *unforeseen event*: one person every two, randomly selected by the computer, will lose the 7 tokens earned in Stage 1 for the work, and will become a *Participant B* (see the picture below). Earnings lost earnings by Participant B will be given to another person, a *Participant A*, that is randomly selected among the 5 members of the Green Group who did not suffer the unforeseen event (see the picture below).

If you are a Green Group member, you will see on your screen the role that was assigned to you. If you are a Red Group member, you will be allowed to surf the Internet.

Stage 4—Decision. Every Participant A is paired with the Participant B from whom he/she received the earnings. Each Participant A will have to decide whether to return the earnings to Participant B or not:

- If he/she decides to *not return*, A earns a total of 12 tokens and B remains with 0 tokens.
- If he/she decides to *return*, A earns a total of 9 tokens and B restores his/her initial earning of 7 coins with a probability between 67 and 100%. When B does not restore his/her earnings, then the 7 tokens are transferred to a member of the Red Group, selected at random. The outcome of the restitution is determined with a die roll (performed by the computer).

Participants’ total earnings are summarized in the following diagram:

The situation just described will be repeated three times. In each repetition, each Participant B will be paired again with a new Participant A, different from the one previously met (i.e. you will never be paired with the same person for more than one repetition). Are there any questions about this?

There are still three important things to say about Stage 4:

1. In each one of the three repetitions, the restoring probability is different and randomly changes between 67 and 100% in the following ways:
  Important: Only Participant A knows the exact probability with which the 7 tokens will be restored by B. Participant B will *never* know it, because the order with which the different restoring probabilities appear is random and remains unknown to B.
2. All the decisions will be taken sequentially, without receiving any feedback on their outcome. At the end of Stage 4, all participants will see the number of tokens they earned. Only Participant A will be informed about the outcome of his/her choice in the repetition randomly selected for the payment (see below).
3. One of the three repetitions, selected at random by the computer, will be paid at the end of the session.

Before proceeding, we ask you to answer some control questions about the instructions. *The experimenter stops reading the instructions, and participants are asked to answer the quiz (Sect.* D*in Appendix). The experimenter privately answered possible participants’ questions. Once every participant has completed the quiz, the experiment restarts reading the instructions.*

Lets now go back to Stage 2.

Stage 2—Estimation. You are now asked to report your expectations about the choices of Participants A in Stage 4 (decision). In particular, you must estimate how many times you expect a generic Participant A will choose to return the earnings to Participant B across the three repetitions (from 0 out of 3, to 3 out of 3). Is that clear?

The more your estimate is accurate, the more money you can earn. To evaluate the accuracy of your estimate, the computer will match you to a Participant A selected at random. The Participant A randomly selected to determine your earnings for Stage 2 (estimate) will be different from the Participant A who will be selected at random to determine your earnings in Stage 4 (decision). At the end of the session, the computer will draw at random one of the three decisions made by Participant A you are matched to. The computer will compare your estimate with the choice of A in the drawn decision (the choice could be either “Return” or “Not return”).

In the table you can see how much you will earn given your estimate and the choice of Participant A.

**Table:**

	Dictator will choose *Return*...			
	0 out of 3	1 out of 3	2 out of 3	3 out of 3
Your guess...	$\Box$	$\Box$	$\Box$	$\Box$
Your earnings if in the drawn choice...				
Dictator chose *Return*	0 €	2.80 €	4.40 €	5 €
Dictator chose *Keep*	5 €	4.40 €	2.80 €	0 €

Note: During Stage 4, before each decision, Participants A will be also informed about the estimate made in Stage 2 by Participant B with whom they are paired.

Payment: The total amount paid at the end of the session (in addition to the €3 for showing up) is the sum of the gain for the accuracy of your estimate in Stage 2, and your earnings in the repetition randomly selected from Stage 4.

From here on, Red Group members can surf the Internet.

#### C.2 Study 2—Workers

Welcome!

You are taking part in a study on economic decision-making. For showing up you earn 3 Euros, and you may, depending on the actions you and others take, earn additional money. Please, follow the instructions carefully, turn off your cellphone, and do not speak to the other participants. If you have questions, raise your hand and one of the experimenters will answer to you in private. All your decisions will remain anonymous.

##### Part 1—Production

In th first part, you will have an assignment on which you have to work for *5 min*. We will measure your performance by the number of points you receive. You will be informed about your score at the end of the assignment.

Description of the assignment

You will be asked to calculate the sum of five randomly chosen two-digit numbers as the one in the example below:

You will be given 5 min to calculate the correct sum of a series of these problems. You cannot use a calculator to determine this sum, however you are welcome to write the numbers down and make use of the provided scratch paper. You can confirm a problem’s result by clicking the OK button with your mouse. When you enter an answer the computer will immediately tell you whether your answer is correct or not.

You will receive 1 point per problem you solve correctly within the 5 min. You do not lose points if you provide an incorrect answer to a problem.

Are there any questions? *Participants work for 5 min on the task.*

##### Part 2—Payments

Now you have completed your work on the assignment, we will now explain how you will be paid.

The computer will match you with another participant who has completed the same assignment. In a few days a third person selected at random will be given the opportunity to assign additional 6 Euros to either you or the other participant you are paired with. The person will not know the identity of neither you nor the other participant, but will be informed about your performance, relative to that of the other participant.

Either you or the other participant obtains the 6 Euros, depending on the choice of the third person. Te participant who does *not* receive the 6 Euros will receive no additional amount. The third person can also choose to assign the 6 Euros at random to you or the other participant by flipping a virtual coin. In this case, both you and the other participant have 50% probability to receive the additional 6 Euros.

In a moment we will ask you to fill an anonymous questionnaire that also includes the two following questions:

1. Who was the best participant in your pair in terms of correctly solved sums? Please indicate whether you were the best in your pair, or whether the other participant was the best.
2. With which probability do you expect that the third person will assign the additional 6 Euros to you? In this question we will reward you for the accuracy of your expectation: the closer your expectation to the realized outcome the higher will be your reward. In particular, we will employ a compensation scheme that ensures that your expected earnings will be at their maximum when you state your true expectation. On your screen you will be able to see how your earnings vary depending on the expectation you state and on the actual outcome. Information on your expectation and that of the other participant can be communicated to the third person.

Are there any questions?

At the end of today session you will receive 3 Euros for participating. On Thursday the 19th of April you can come to the CEEL to receive the payment related to the third person choice and to your expectations. You can come whenever you prefer between 9:30 am and 12:00 am. To identify yourself we ask you to insert the last 4 digits of your cellphone in a scree and this will be your identifier. To receive the payment you will need to bring your cellphone linked to your identifier.

Your expectation and those of the participant you are matched with might be communicated to the third person.

#### C.3 Study 2—Spectators

Welcome!

You are taking part in a study on economic decision-making. For showing up you earn 7 Euros. The total amount you earned will be paid to you in private at the end of this session. Please, follow the instructions carefully, turn off your cellphone, and do not speak to the other participants. If you have questions, raise your hand and one of the experimenters will answer to you in private.

In this study you will be asked to make choices that have actual consequences on the earnings of other people.

A few days ago we recruited some individuals to work on the same task at the CEEL laboratory. Afterwards, we matched them in pairs. We will call the two members of each pair worker A and worker B. Both workers received a participation compensation of 3 Euros regardless of their work performance. After completing their work, A and B were *not* informed about who was the most productive worker. However, they were told that a third person, i.e. you, would be informed about the assignment and who was the most productive worker. A and B also knew that the third person would be given the opportunity to assign additional 6 Euros to one of the two and thus determine how much they were paid for the assignment.

The workers were also asked to state their personal expectation of being the worker chosen by the third person to obtain the additional 6 Euros. Workers had an economic incentive to state their expectation in an accurate way.

You are the third person and we now ask you to choose whether to assign the 6 Euros to worker A or worker B. Alternatively, you can flip a virtual coin to determine who will receive the 6 Euros between A and B. Each worker has 50% probability to obtain the 6 Euros. At the moment of your choice you will be informed on who between the two workers was more productive and who has higher personal expectations of obtaining the additional 6 Euros.

In total, you will have to make 20 choices that correspond to the 20 pairs of workers A and B who performed the task. At the end of this study we will randomly select one of the individuals who participated as a third person. The choices of the selected third person will determine the payment of *all* the pairs of workers. If you will be selected for the payment of the workers, in everyone of the 20 pairs for which you made a decision, the worker that you selected will receive the payment of 6 Euros. Moreover, the worker will be informed of whether he or she received the 6 Euros as the random outcome of your coin flip or because you assigned the 6 Euros to him or her. Your decisions will remain anonymous.

After you made your decisions we will ask you to fill an anonymous questionnaire.

Are there any questions?

#### C.4 Robustness check—Workers

You are taking part in a study on decision-making that consists of four stages.

You earn a fixed amount of money for participating in the study.

You may earn an additional bonus payment. The size of the bonus payment will be affected by choices of others and by a random draw. The earnings are expressed in Pound sterling (£).

We will pay your bonus payment after all other participants have answered the study.

All your choices are anonymous.

Please, read the instructions carefully.

##### Stage 1—Work

—

*For Subject C only*

You are a Subject C. These intructions refer to Worker A and B, you will not count the zeroes and will move directly to the Stage 2—estimation. However, we invite you to read the instructions to understand next stages.

—

In this stage you must count the number of zeros in seven tables that will consecutively appear on your screen. For each table you will earn £1, once you input the correct number of zeros.

This is an example of the table you will be asked to count

The number of zeroes for this specific table is 36.

The number of zeroes will randomly change in the seven tables you are facing in Stage 1.

—

*For Worker A and B only*

In the next screen you will start counting the zeroes in the tables. Afterward, something unforeseen could occur and you might lose the £you earned. You will receive more details about this event after you finished counting.

—

To understand Stage 2 (estimation), you first need to learn about Stage 3 (unforeseen event) and Stage 4 (decision). Before proceeding to Stage 2, we anticipate you the instructions for Stages 3 and 4.

##### Stage 3—Unforeseen event

In this stage, you do not take any decisions. The computer will implement the procedures.

You could suffer an unforeseen event: the computer could randomly select you to lose the £7 you earned in Stage 1 for your work.

If this happens to you we will call you a Worker B (see the picture below).

Earnings lost by Worker B will be transfered to another participant, a Worker A, who participated in Stage 1 (work) just like you did, but did not suffer the unforeseen event (see the picture below).

You will have the same probability of becoming Worker A or Worker B

##### Stage 4—Decision

A third person—who did not participate in Stage 1 (work) and has no direct monetary stake in the choice—can decide whether to return the earnings to Worker B or not (see figure below):

(1) If he/she does not return, Worker A earns £12 and Worker B earns £0. (2) If he/she returns, Worker A earns £9, but Worker B restores his/her reward of £7 only up to a certain restoring probability, that can range between 67 and 100%. The outcome of the restitution in this case is determined with a die roll performed by the computer. When Worker B does not restore his/her reward, the £7 reward is misplaced to an Individual C, who did not do any work.

The diagram below summarizes participants’ earnings. All participants in this study know this earnings structure.

There is still one important things to say about Stage 4: the restoring probability faced by the third person can take one of three values: 67% (4 out of 6), 83% (5 out of 6), or 100% (6 out of 6). The third person has exactly the same likelihood of of facing each one of the three alternative restoring probabilities.

You can think of this probability as the likelihood, when tossing a fair 6-sided die, of getting a number smaller or equal than 4, 5, and 6, respectively.

Important: Only the third person knows the exact probability with which the 7 tokens will be restored by B. Workers A and B, as well as individual C, will never know it.

##### Stage 2—Estimation

You are now asked to report your expectations about the behavior of the third person in Stage 4 (decision). In particular, you must estimate the likelihood with which you expect a generic third person to choose to return the earnings to Worker B. You can express your estimate by choosing one of four intervals of likelihood: 0–25%, 26–50%, 51–75%, 76–100%.

Remember that third persons will make choices for each level of restoring probability: 67% (4 out of 6), 83% (5 out of 6), or 100% (6 out of 6).

The more accurate your estimate, the more money you can earn.

To define your bonus payment, we will follow this procedure: (1) We will collect the return choices of all third persons, for all three levels of restoring probability. (2) One return choice of the third person will be randomly drawn. (3) The randomly drawn choice of the third person will be compared with your estimate. (4) The matching between your estimate and the choice of the third person will define your earnings, as from the table below.

**Table:**

	Probability that a third person will choose Return			
	0–25%	26–50%	51–75%	76–100%
Your earnings if a third person	If you choose this	If you choose this	If you choose this	If you choose this
Chooses Return	£0.00	£0.28	£0.44	£0.50
Does not choose Return	£0.50	£0.44	£0.28	£0.00

Note: During Stage 4, the third person will be also informed about the estimate made in Stage 2 by Workers B.

#### C.5 Robustness check—Spectators

You are taking part in a study on decision-making that consists of two parts: Part 1 and Part 2.

You earn a fixed amount of money for participating in Part 1.

You may earn an additional bonus payment in Part 2. The size of the bonus payment in Part 2 will be affected by your choices and choices of others.

We will pay your bonus payment after all other participants have answered the study.

All your choices are anonymous.

Please, read the instructions carefully.

##### Part 1

You will make decisions that can have an actual impact on the earnings of other people. The earnings are expressed in Pound sterling (£).

In another session of this study we recruit two individuals to work on the same task. Both individuals obtain a reward of £7 for their work. However, after the task one of the two individuals is selected at random and loses his/her reward. We call this individual Worker B. The reward lost by Worker B is transferred to the other individual, who did not lose his/her reward. We call this other individual Worker A.

Workers A and B are told that a third person can decide whether or not to return the reward to Worker B. You are this third person and we ask you to choose whether or not to return the reward to Worker B:

- If you do not return, Worker A earns £12 and Worker B earns £0.
- If you return, Worker A earns £9, but Worker B restores his/her reward of £7 only up to a certain restoring probability, that can range between 67 and 100%. The outcome of the restitution in this case is determined with a die roll performed by the computer. When Worker B does not restore his/her reward, the £7 reward is misplaced to an Individual C, who did not do any work.

The diagram below summarizes participants’ earnings. All participants in this study know this earnings structure.

There are three more important things to say:

1. The restoring probability that you will face can take one of three values: 67%, 83%, or 100%. Each of these values has the same likelihood of being selected. The value randomly selected for you is: DISPLAYED LAMBDA
  You can think of this probability as the likelihood of getting a number smaller or equal than DISPLAYED LAMBDA when tossing a fair 6-sided die.
  [ONLY THE PICTURE CORRESPONDING TO THE DISPLAYED LAMBDA IS SHOWN]
  Important: You are the only participant to know the exact restoring probability. Workers A and B know that you are facing one of the three equally likely restoring probabilities, but they do not know which one.
2. Before informing Worker B that he/she has lost her reward, we ask Worker B to state his/her expectations about the likelihood for a third person to return the reward.
  We ask you to take four distinct return decisions, one for each possible level of expectation that Worker B can have: 0 to 25% probability that a third person returns the reward, 26 to 50% probability that a third person returns the reward, 51 to 75% probability that a third person returns the reward, 76 to 100% probability that a third person returns the reward.
  Note that you don’t know the actual level of expectation of Worker B.
  The following is an example of the choice you are going to make. Choices are made by selecting the option you prefer.
  **Table:**
  #	Expectations of Worker B (%)	Your return decision
  1	0–25% that a third person chooses to return	O Return; O Not return
  The choice on this page has no consequences on your actual earnings.
  At the end of the study, one individual who participated in the role of third person will be selected at random. The earnings of Workers A and B will depend on the decision made by the selected third person in correspondence of the actual level of expectation stated by Worker B.

##### Part 2

You will see again the four return choices you just saw in Part 1.

For each of the choices, you will be asked to judge the social appropriateness of each Option A, relative to Option B. You can choose among 4 levels of social appropriateness:

- Very inappropriate
- Somewhat inappropriate
- Somewhat appropriate
- Very appropriate

When evaluating the social appropriateness, please refer to the restoring probability you faced: DISPLAYED LAMBDA

The following is an example of the choice you are going to make. Choices are made by selecting an option from the dropdown menu.

**Table:**

#	Expectations of Worker B (%)	Social appropriateness of Return
1	0–25% that a third person chooses to return	Very inappropriate; Somewhat inappropriate; Somewhat appropriate; Very appropriate

The choice on this page has no consequences on your actual earnings.

On the next page, you will make a choice for each of the four expectation levels you faced in Part 1. Choices are made in a table, and each row contains a choice among the four social appropriateness levels.

At the end of the session, one of the four choices will be randomly selected for payment.

For the randomly selected choice, the payment is computed as follows:

- Choices of all participants in the session who faced the same restoring probability you faced are counted, and the social appropriateness level chosen by the majority is computed;
- If your choice is the same as that chosen by the majority, you earn an additional £0.5 (50p); if your choice is not the same as that chosen by the majority, you earn £0.

### D Slides and quiz of Study 1

#### D.2 Translation of the quiz

1. During Stage 3, how are roles assigned to Green Group members?
  - At random.
  - Those who have not worked well suffer the unforeseen event and become Participants B.
  - Those who have not worked well suffer the unforeseen event and become Participants A.
2. During Stage 4, every Red Group member...
  - chooses whether or not to return the earnings lost by Participant A.
  - leaves the room.
  - waits for all Participants A to complete their choices, surfing the Internet.
3. During Stage 4, every Participant B...
  - chooses whether or not to return the earnings lost by Participant A.
  - waits for all Participants A to complete their choices.
  - waits for all Participants A to complete their choices, surfing the Internet.
4. During Stage 4, every Participant A...
  - chooses whether or not to return the earnings lost by Participant B.
  - waits for all Participants A to complete their choices.
  - waits for all Participants A to complete their choices, surfing the Internet.
5. During Stage 4, what happens when a Participant A chooses to return the earnings to Participant B?
  - The earnings are always restored by Participant B (the original owner).
  - A die is rolled to establish whether the earnings actually are restored to B. The restoring probability is always different.
  - A die is rolled to establish whether the earnings actually are restored to B. The restoring probability is always the same.
6. During Stage 4, what happens when a Participant A chooses to return the earnings to Participant B but the roll of the die determines that the earnings are not restored?
  - The earnings are not returned to anybody and thus it is lost.
  - The earnings remain to Participant A.
  - The earnings are transferred to a member of the Red Group chosen at random.
7. In each repetition, the restoring probability...
  - is known to all participants.
  - is known only to Participant B.
  - is known only to Participant A.
8. When is the die rolled?
  - Whenever Participant A chooses to not return.
  - Whenever Participant A chooses to return.
  - Always, irrespective to the choice of A.
9. Participant A...
  - cannot change her decision (“Return”/“Not Return”) across the three repetitions.
  - can change her decision (“Return”/“Not Return”) across the three repetitions.
  - cannot change her decision (“Return”/“Not Return”) in only one repetition.

## Footnotes

1 As in other experiments testing guilt aversion, we focus on a binary decision rather than allowing *D* to choose an amount to return. This feature restricts the decision-makers’ action space relative to a continuous specification. However, a decision-maker can still choose the action that better meets her motivations.

2 The higher valuation of *ER* for her wallet could be interpreted as some intrinsic value she attaches to it. This interpretation, however, is less suitable to justify payoffs when the wallet is misplaced: realism would require *UR* and *D* to give the same value to *ER*’s wallet.

3 Different $\lambda$ values capture different degrees of protection to the entitlement right of *ER* due to exogenous institutional aspects.

4 Interactions between groups *A* and *B* were avoided. Group *B* joined the session 10 min later and left earlier than group *A*. We let group *B* members surf the Internet to highlight their lack of effort and entitlement on the lost wallet.

5 We adopted this real-effort task by Abeler et al. (2011) because it is arguably more related to effort provision than knowledge tests (used, for instance, by Cherry et al. 2002) in which human capital and luck are key. Different from Abeler et al. (2011), all group *A* members ended up with the same endowment. We did this to ease the empirical analysis. We believe that the task successfully conveyed a sense of entitlement to group *A* for three reasons. First, in the instructions (see Sect. C.1 of the Appendix), group *A* members were clearly told that their earnings were a piece-rate reward for each table they *had to* solve (we did not use the word wallet). Second, the task lasted, on average, 10 min, a non-trivial fraction of the duration of a session (roughly 1 h). Third, during the real-effort task, group *B* members were not yet in the lab.

6 This design choice might raise the concern that group *A* members could strategically manipulate their expectations. However, we carefully designed the belief elicitation to discourage such behavior. First, subjects had a monetary incentive to reveal their true expectations. Second, expectations were elicited before roles were assigned to subjects. Finally, we can control for this issue ex-post, by comparing the expectations’ distributions of groups *A* and *B*; group *B* members knew their expectations were not going to be disclosed to dictators.

7 The average age of participants is 23 years, females are 49.4% of the sample, and 90% of participants have Italian citizenship. About half of the sample (56.1%) is made of students of Economics, and most of the participants do not actively work (68.3%).

8 If *D* is efficiency concerned, she chooses Return because this maximizes total earnings regardless of $\lambda$. Under the assumption of inequity aversion, *D* also opts for Return regardless of $\lambda$ because this minimizes the inequality. The same prediction holds under a procedural version of the model (Trautmann 2009). Taking the expected value of the Return lottery generates an even fairer allocation than in the standard consequentialist framework. Thus, unlike in other settings (e.g., Krawczyk and Le Lec 2010), uncertainty does not affect inequality predictions. Finally, pure altruism also leads *D* to always choose Return.

9 Gill and Stone (2010) argue that when one gets more than what is deserved, she can either experience a welfare gain (desert elation) or a welfare loss (desert guilt). However, when the other gets less than what is deserved, a loss of welfare is unambiguously experienced (desert loss). Moreover, they assume that welfare changes when receiving less than what deserved are larger than changes in welfare when receiving more as a result of loss aversion.

10 We neglect the expectation level “Always” because only one observation is available. The number of independent observations for the other expectation levels are as follows: 19 (31.7%) for “Never”, 27 (45.0%) for “Seldom”, and 13 (21.7%) for “Often”.

11 For any $\lambda$, the comparisons of “Never” and “Seldom”, and of “Seldom” and “Often” do not reveal any significant difference ($p\ge 0.115$).

12 In an exploratory step, we also estimated a linear model. Results are consistent with those from the generalized linear model reported here and are available upon request.

13 Variables relating to the dictator’s own expectation may suffer from endogeneity, which makes it hard to give a causal interpretation of the results.

14 We cannot fully rule out that dictators suspected that entitled recipients manipulated their expectations (see footnote 6). If this were the case, they would be arguably less likely to fulfill them. Thus, we take this effect as a lower bound.

15 We thank an anonymous referee for suggesting this additional study.

16 We implemented the experiment online because the CEEL laboratory at the University of Trento was temporarily closed due to the COVID-19 pandemic. We minimized differences between the online sample and Study 1 sample by restricting participation only to students between 18 and 30 years old. Females were 42.7% of the sample, and the average age was 27. Subjects were only allowed to take part in the experiment using a computer or a tablet and not a smartphone. We could not restrict participation to Italians only because this additional requirement was too restrictive.

17 Note that *ER*’s expectations in Study 1 referred to the number of return decisions of *D*, knowing that *D* was facing three levels of $\lambda$. Given that in the robustness check each external spectator faced only one $\lambda$ level, we could not elicit the same type of expectation. Thus, we elicited expectations over four non-overlapping equally-spaced intervals that referred to the frequency of returns for the entire population of external spectators.

18 The payoff, in Euro, for the belief task is given by $S_i(p)=2-\frac{1}{10,000}\sum _{k=1}^2 (I_k-p_k)^2$, where *I* is equal to 1 when the event *i* is realized and equal to zero otherwise. As an example, when a worker reported a 70% probability of being rewarded, he/she would earn €1.82 when actually rewarded and €1.02 when not rewarded. The earnings for all combinations of probabilities and outcomes were reported on the computer screen, but the underlying function was not to avoid potential confusion. All workers had to come back to the lab two days after the session to collect their payment for the accuracy of their first-order beliefs, and, possibly, the reward of €6.

19 Pairs of workers were randomly formed at the spectator-level. We did not disclose the type of task that the workers performed.

20 Workers knew about this possibility, and spectators, in turn, knew that the worker who obtained the €6 would learn whether this was the outcome of a deliberate decision or of the coin flip.

21 The following frequencies of alternative combinations are empirically observed: better performance/higher expectation (52.8%), better performance/lower expectation (20.0%), better performance/same expectation (16.3%), same performance/higher expectation (6.8%), same performance/same expectation (4.2%).

22 We adopted the Italian translation of TOSCA-3 by Anolli (2010).

23 Choices of spectators are very consistent at the individual level, with only 16.7% choosing differently across repetitions for conditions not involving the same level of expectation and performance.

24 To perform the tests, we compare the average choice of a spectator in a given condition with the average choice of the same spectator in a different condition. This way, we circumvent dependency issues in the data due to repeated choices. We omit from the analysis the conditions involving the same relative performance and the same relative expectations, as the number of observations available is too low.

25 In a series of exploratory regressions, we distinguished between better, equal, and worse performance and between higher, equal, and lower beliefs. We decided to choose the specification reported here, which pools together equal and lower expectation and equal and worse performance, because of the few observations available for equal levels of the two variables in our couples. The main results do not change across specifications.

26 The TOSCA-3 questionnaire we administered delivered information also about shame, pride, externalization, and unconcern. We focus here only on the variable of our interest, i.e., guilt.

27 A series of linear hypothesis tests show that the estimated coefficients of *PerfBetter* and of *ExpHigher* are significantly different across all specifications ($\chi ^2$ test, $p<0.001$).

28 In an exploratory step, we also estimated a linear model. Results are overall consistent with those obtained from the generalized linear model reported here and are available upon request.
